# Efficacy of a physiotherapy rehabilitation program for individuals undergoing arthroscopic management of femoroacetabular impingement – the FAIR trial: a randomised controlled trial protocol

**DOI:** 10.1186/1471-2474-15-58

**Published:** 2014-02-26

**Authors:** Kim L Bennell, John M O’Donnell, Amir Takla, Libby N Spiers, David J Hunter, Margaret Staples, Rana S Hinman

**Affiliations:** 1 The University of Melbourne, Centre for Health, Exercise and Sports Medicine, Department of Physiotherapy, School of Health Sciences, Melbourne, Vic, Australia; 2 St Vincent’s Private Hospital, Melbourne, Vic, Australia; 3 Ivanhoe Physiotherapy Clinic, Melbourne, Vic, Australia; 4 Royal North Shore Hospital, Rheumatology Department, Sydney, NSW, Australia and Kolling Institute, University of Sydney, Sydney, NSW, Australia; 5 Department of Clinical Epidemiology at Cabrini, Monash University, Melbourne, Vic, Australia

**Keywords:** Physiotherapy, Physical therapy, Rehabilitation, Hip arthroscopy, Femoroacetabular impingement

## Abstract

**Background:**

Femoroacetabular impingement is a common cause of hip/groin symptoms and impaired functional performance in younger sporting populations and results from morphological abnormalities of the hip in which the proximal femur abuts against the acetabular rim. Many people with symptomatic femoroacetabular impingement undergo arthroscopic hip surgery to correct the bony abnormalities. While many case series over the past decade have reported favourable surgical outcomes, it is not known whether formal rehabilitation is needed as part of the management of patients undergoing this surgical procedure. This randomised controlled trial will investigate the efficacy of a progressive physiotherapist-supervised rehabilitation program (Takla-O’Donnell Protocol) in improving health-related quality of life, physical function and symptoms in individuals undergoing arthroscopic management of femoroacetabular impingement.

**Methods/design:**

100 people aged 16–35 years undergoing hip arthroscopy for symptomatic femoroacetabular impingement will be recruited from surgical practices in Melbourne, Australia and randomly allocated to either a physiotherapy or control group. Both groups will receive written information and one standardised post-operative physiotherapy visit whilst in hospital as per usual care. Those in the physiotherapy group will also receive seven individual 30-minute physiotherapy sessions, including one pre-operative visit (within 2 weeks of surgery) and six post-operative visits at fortnightly intervals (commencing two weeks after surgery). The physiotherapy intervention will incorporate education and advice, manual techniques and prescription of a progressive rehabilitation program including home, aquatic and gym exercises. The control group will not receive additional physiotherapy management. Measurements will be taken at baseline (2 weeks pre-operatively) and at 14 and 24 weeks post-surgery. Primary outcomes are the International Hip Outcome Tool and the sports subscale of the Hip Outcome Score at 14 weeks post-surgery. Secondary outcomes include the Copenhagen Hip and Groin Outcome Score, the activities of daily living subscale of the Hip Outcome Score, the Heidelberg Sports Activity Score, a modified Tegner Activity Scale and participant-perceived overall change.

**Discussion:**

The findings from this randomised controlled trial will provide evidence for the efficacy of a specific physiotherapist-supervised rehabilitation program in improving outcomes following arthroscopic management of symptomatic femoroacetabular impingement.

**Trial registration:**

Australian New Zealand Clinical Trials Registry reference number: ACTRN12613000282785.

## Background

Femoroacetabular impingement (FAI) is a common cause of hip/groin symptoms and impaired performance in younger sporting populations [1-3]. FAI is the result of morphological abnormalities of the hip in which the proximal femur abuts against the acetabular rim. This impingement is due either to abnormalities in the morphology of the femoral head (referred to as cam impingement) and/or excessive acetabular coverage of the femoral head (referred to as pincer impingement) [4]. Not only can FAI give rise to symptoms and impair function, the repetitive inappropriate bony contact can also lead to a cascade of structural damage including tearing at the chondrolabral junction, full thickness cartilage delamination and eventually hip osteoarthritis [5,6].

Hip arthroscopy is often used in the assessment and management of hip pathology. Indeed the total number of hip arthroscopies performed around the world is rapidly increasing [7]. Currently, post-operative management of patients following hip arthroscopy for FAI is variable, and dependent on surgeon’s preferences and patient access to rehabilitation services. Some patients undergo formal physiotherapist-supervised rehabilitation, whilst others do not. Physiotherapist-supervised rehabilitation is advocated in the literature in order to restore muscle function and strength and improve joint range of motion, as well as facilitate a safe and graded return to sporting activity. Several rehabilitation protocols following hip arthroscopy have been described in the literature [8-14]. These impose varying post-operative restrictions related to weight-bearing and hip range of motion, and utilise different exercise and therapeutic techniques; however there is no high quality evidence that any one is more effective than another, or indeed, more effective than no formal post-operative rehabilitation.

Due to the dearth of research in this area, evidence-based recommendations to guide the post-operative management of patients following hip arthroscopy for FAI cannot be made. Accordingly, based on their extensive clinical experience, two of our team members (AT physiotherapist and JOD orthopaedic surgeon) developed a specific physiotherapist rehabilitation program (Takla-O’Donnell Protocol (TOP)), designed to facilitate return to sport typically within three months of surgery. Whilst anecdotally this protocol appears effective, it has not been subjected to formal research evaluation. Thus, the objective of this randomised controlled trial (RCT) is to evaluate the efficacy of the TOP, a progressive physiotherapist-supervised rehabilitation intervention, on health-related quality of life, physical function and symptoms in individuals undergoing hip arthroscopic management of FAI. The primary time point will be measured at 14 weeks post-surgery, a time when typically patients would have completed the TOP rehabilitation and returned to their usual activities.

Our primary hypothesis is that in individuals undergoing hip arthroscopy for symptomatic FAI, those in the physiotherapy group (PT) will report significantly greater improvements in health-related quality of life, as measured by the International Hip Outcome Tool (iHOT-33), and function in sport, as measured by the sports subscale of the Hip Outcome Score (HOS), at 14 weeks post surgery than those in the control group (CON) not undergoing formal rehabilitation.

## Methods/design

### Trial design

We will conduct a parallel-design 2-arm RCT with outcomes assessed at baseline (within 2 weeks prior to surgery), at 14 weeks post surgery (immediately following the PT intervention) and at 24 weeks post-surgery, with the primary outcome time point being 14 weeks post surgery. Reporting of the study will conform to CONSORT guidelines for non-pharmacological studies [15].

### Participants

100 men and women aged 16–35 years with symptomatic FAI who are scheduled for hip arthroscopy will be recruited from the surgical practices of five orthopaedic surgeons in metropolitan Melbourne, Victoria, Australia.

People will be eligible if they have (i) had hip/groin symptoms for at least 3 months; (ii) been diagnosed with FAI by an orthopaedic surgeon based on symptoms, clinical signs and imaging findings; and (iii) are scheduled for hip arthroscopy.

People will be excluded from participating if they (i) have radiographic evidence of hip osteoarthritis that is more than mild in severity defined as Tonnis > grade 1 [16]; (ii) are a professional athlete; (iii) have other concurrent injury/conditions that will affect their ability to participate in the rehabilitation program and/or assessment procedures; (iv) are unable to attend a study physiotherapist or participate in the rehabilitation program if randomised to the PT group; (v) wish to undertake formal supervised rehabilitation following hip arthroscopy; and (vi) are unable to understand English.

Ethical approval has been obtained from the University of Melbourne Human Research Ethics Committee (HREC No. 1238190). All participants will provide written informed consent.

### Study procedure

**Figure 1:**
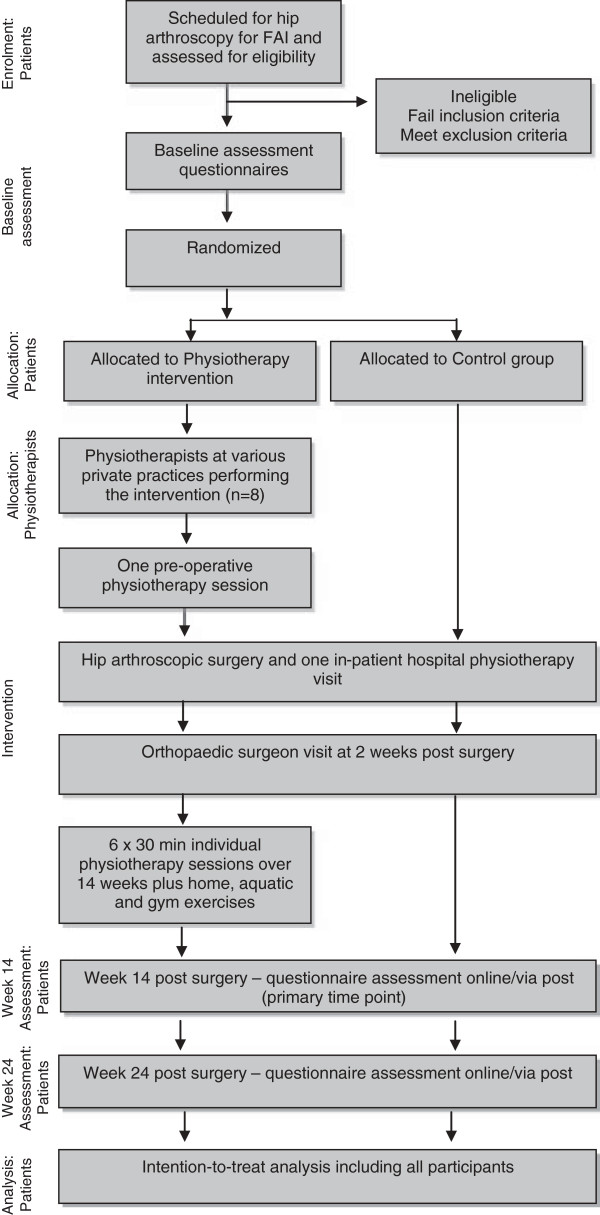
Flow diagram of the study protocol.

Patients who are scheduled for hip arthroscopic surgery and fulfil the eligibility criteria will be identified by the surgeon and provided with study information by staff at the surgeon’s practice. An independent research assistant will confirm eligibility via subsequent telephone screening. Consenting participants will complete baseline questionnaires electronically or via post approximately two weeks prior to surgery. Upon receipt of baseline data, participants will be consecutively randomised into either the PT or the CON group by an independent person not involved in recruitment, assessment or treatment of participants. Participants in both groups will undergo hip arthroscopy for management of their FAI as scheduled by their surgeon and will receive standardised pre- and post-operative care, including an in-patient physiotherapy visit, provision of written educational material and a follow-up appointment with the orthopaedic surgeon at approximately two weeks post surgery. The PT group will additionally receive seven individual 30-minute physiotherapy sessions including one pre-operative session and six post-operative sessions at fortnightly intervals commencing two weeks after surgery. The PT group will also perform a home, aquatic and gym rehabilitation program. Re-assessment will occur at 14 weeks (following completion of physiotherapy rehabilitation in the PT group) and 24 weeks post surgery via administration of questionnaires completed online or via post. A flow chart outlining the study procedures is shown in Figure 1.

### Blinding

It is not possible to blind participants in this study. The study physiotherapists treating participants in the PT group will be, by necessity, unblinded. The researcher managing the patient-completed data will be blinded to group allocation as will the physiotherapists providing in-patient treatment to both groups. The orthopaedic surgeon will also be blinded to group allocation and participants will be asked not to disclose this to the surgeon at their follow-up appointment. The statistician will be blinded to group allocation until completion of the statistical analyses.

### Randomisation and allocation concealment

The randomisation schedule will be prepared by the study biostatistician using a computer-generated random numbers table. There will be a 1:1 allocation ratio of participants to the PT and CON groups. Randomisation will be conducted by random permuted blocks of varying size, and stratified by orthopaedic surgeon (so that each surgeon contributes approximately equal numbers in each group to control for surgical variation) as well as by whether the participant is having unilateral or bilateral surgery. Participants in the PT group will choose their preferred project physiotherapist according to geographical convenience.

Consecutively numbered, sealed, opaque envelopes containing group allocation will be prepared by a researcher with no other involvement in the study. The envelopes will be stored in a locked location and will be opened in sequence to reveal group allocation by a researcher not involved in recruitment, treatment or assessment of outcomes.

### Interventions

All participants will receive hip arthroscopic surgery for FAI performed by an experienced hip orthopaedic surgeon. Unstable articular cartilage flaps will be debrided (chondroplasty) and any exposed subchondral bone will be treated by microfracture if its area is <400 sq mm. Superficial labral tears will be debrided and tears that are unstable or >50% deep will be repaired. No segmental labral resections will be performed. Partial or complete tears of the ligamentum teres will be debrided with a radiofrequency probe. Cam impingement lesions will be treated by femoral osteochondroplasty. Pincer impingement lesions will be treated when there is both radiological and intra-operative pathological evidence to confirm the diagnosis. At the end of the procedure the operative field will be injected with 5mgs Morphine, 30mgs Ketorolac and 20 ml of 0.2% Naropin.

Immediate postoperative care at St Vincent’s Private Hospital East Melbourne and Bellbird Private Hospital will be consistent for both groups. Patients will stay in hospital overnight, unless they specifically request to leave on the same day. Ice and compression will be applied to the hip overnight. Standard care will include (i) an in-patient physiotherapy visit for provision of a gait aid; (ii) the surgeons’ usual inpatient written educational material covering post-operative precautions, return to activity and basic hip exercises; and (iii) use of a non-steroidal anti-inflammatory drug, as selected by the surgeon, for one month as prophylaxis against heterotopic bone formation in those without contraindications to their use. A follow-up visit with the orthopaedic surgeon will occur at approximately two weeks post surgery.

Patients will be asked to use crutches until they can walk without pain and without limping, likely 10 days or less. Patients will be advised to avoid hip flexion past 90 degrees for approximately six weeks and to avoid positions that have the potential to cause impingement and increase inflammation. This will include avoidance of deep squatting, lifting heavy objects from the floor, pivoting or twisting on a fixed foot, sitting with the hip flexed past 90 degrees, as well as prolonged sitting.

#### Physiotherapy

Participants in the PT group will attend a study physiotherapist for one pre-operative visit (after baseline assessment) within two weeks prior to surgery, and six post-operative visits on a fortnightly basis commencing at week two (approximately 2, 4, 6, 8, 10 and 12 weeks post surgery). Each session will be a 30-minute individual appointment. Physiotherapists who have at least two years of musculoskeletal experience and work in private clinics in metropolitan Melbourne and regional Victoria will be trained to provide the physiotherapy intervention.

The physiotherapists will follow a progressive semi-structured program based on the Takla-O’Donnell Protocol, a clinical protocol developed and refined by two of the authors over a 10-year period. It will comprise of standardised assessments/re-assessments, education and advice, manual therapy techniques, prescription and progression of a home, aquatic and gym program, and graduated return to sport and physical activity. A summary of the physiotherapy intervention is provided in Tables 1, 2, 3, 4. Participants will receive handouts demonstrating the home exercises as well as a log-book to record completion of home, aquatic and gym sessions.

**Table 1:** The physiotherapy intervention – manual therapy techniques

**Manual Therapy Techniques**	**Aim**	**Description**	**Timeframes**	**Dosage**
*Mandatory technique:*				
Trigger point massage of rectus femoris, adductors, tensor fascia latae/gluteus medius/gluteus minimus and pectineus muscles and associated fascia	To address soft tissue restrictions with the aim of reducing pain and improving hip range of movement	Sustained pressure trigger point release with the muscle on stretch. In general, mobilise restrictions laterally to the line of tension of the muscle being treated	Session 2-7	30-60 seconds per trigger point
*Optional technique:*				
Lumbar spine mobilisation, if indicated by lumbar spine physiotherapy assessment	To improve mobility and pain-free movement of the lumbar spine to assist with hip function	Unilateral postero-anterior accessory glides, Grade III or IV	Session 3-12	3-5 sets of 30–60 seconds

The treatment program is semi-structured, and includes a number of mandatory components plus some optional components. Individual program progression will be guided by assessment findings and the nature of the surgical procedure.

**Table 2:** Home exercises

**Home exercises**	**Aim**	**Description**	**Timeframes**	**Dosage**
Deep hip rotator muscle retraining (see Additional file 1)	Optimise hip neuromuscular control and improve dynamic stability of the hip	Seven stages progressing through prone, four-point-kneel and dynamic standing positions, with and without additional resistance.	Pre-op to session 7	1 minute, 3–6 times per day
Anterior hip stretch	Assist in regaining full hip extension range of movement	Supine in modified Thomas Test position with the affected leg over the side of the bed. The hip is extended until a stretch is felt at the front of the hip	Session 2 - 4	5 minutes daily
Hip flexion/extension in four-point kneel – “pendulum” exercise	Prevent adhesions, especially in those with labral repair	Four point kneel with gentle pendular swing of the affected leg into hip flexion and extension as far as comfortable	Session 2- 5	1 minute daily
Posterior capsule stretch		Lying on unaffected side with the affected hip as close to 90 degrees flexion as comfortable and affected leg over the side of the bed.	Session 3 – 7 (or session 4 – 7 if microfracture present)	3 × 30 seconds

**Table 3:** Gym/aquatic program

**Gym/aquatic program**	**Aim**	**Description**	**Timeframes**	**Frequency**
Stationary cycling	To improve hip range of motion	Upright bike with high seat to avoid hip flexion past 90 degrees. Initially 15 mins at moderate intensity	Session 2 onwards (Session 3 if have microfracture)	2 × weekly
Walking in pool	To maintain cardiovascular fitness and improve hip range of motion	Walking at chest depth, forwards, straight lines only. 10 mins for FOC or labral repair, 5 mins for microfracture or ligamentum teres repair	Session 2 onwards (session 3 if have microfracture)	2 × weekly
Swimming	To maintain/regain cardiovascular fitness	No kicking until 6–8 weeks post-surgery, 500 m – 1 km	Session 2 onwards (session 3 if have microfracture)	2 × weekly
Cross trainer	To maintain/regain cardiovascular fitness	Initially 15mins at moderate intensity	Session 2 onwards (Session 3 if have microfracture)	2 × weekly
Squats	To improve lower limb strength and function	3 sets of 10 repetitions, working at “moderately hard” on modified Rating of Perceived Exertion (RPE)	Session 6 onwards	2 × weekly
Lunges				
Leg press				
Leg extensions				
Hamstring curls				

**Table 4:** Functional program

**Functional program**	**Description**	**Timeframes**	**Dosage**
Jogging	Jogging on running track or grass, with affected leg to the outside of the track ie anticlockwise for the right hip. One lap of oval should be approx 400 m.	Session 4 onwards for FOC (femoral osteochrondroplasty) only, session 5 onwards for others	3 × weekly 6 laps in first week, 8 laps in second week, 10 laps in third week (ie building up to 4 km)
Acceleration/ change of direction drills	Zig-zag jogging	Session 5 (FOC only)	Dependent on sport goals and surgical procedure
		Session 6 all others	
Sport-specific drills	Examples: foot drills/serving practice (tennis); corner hit-outs/tackling drills (grass hockey); kicking/marking drills (Australian Rules Football)	Session 4 (FOC only)	Dependent on sport goals and surgical procedure
		Session 6–7 all others	

##### Education

Education and advice will be a focus of the pre-operative treatment session as well as an important aspect of the first post-operative session. This will include information regarding post-operative joint protection (such as activity avoidance or modification), return to driving and work, and the importance of the home exercise program.

##### Manual therapy

Manual therapy techniques will be used throughout the rehabilitation program. Trigger point massage will be used at each post-surgical treatment session to release muscle tension, assist with pain relief and improve hip range of motion [17]. Lumbar spine mobilisation, in the form of passive accessory intervertebral movements, will be performed [18] in those patients where the physiotherapy assessment determines it is required.

##### Deep hip rotator muscle strengthening

A key component of the home program is local stabilization of the hip joint by retraining and strengthening the deep hip rotator muscles. This deep musculature includes quadratus femoris, the gemelli, and obturator internus. These muscles have a short lever arm and therefore have the potential to act as deep stabilizers, to steady the femoral head in the acetabulum. It has been suggested that they may provide fine control of hip joint stability, acting as the “rotator cuff” of the hip joint [19,20]. There is some evidence that these deep muscles contribute to dynamic hip stability [21,22] and therefore it is possible that retraining and strengthening of this group may accelerate rehabilitation post hip arthroscopy.

Deep hip rotator muscle retraining follows seven stages, with the participant moving to the next stage once they achieve effective activation and endurance of the deep hip rotators required at that particular stage as determined by the therapist. Exercise sheets provided to study participants show these stages in more detail [see Additional file 1]. Retraining commences pre-operatively in prone, followed by progression to 4-point-kneeling, the addition of resistance band and finally weight-bearing with visual feedback and global muscle recruitment.

##### Stretches and range of motion exercises

Pendular exercise (hip flexion/extension) in 4-point-kneel is included from two to ten weeks post-surgery, to reduce the risk of adhesions. Anterior hip joint capsule stretch (supine with the leg over the edge of a bed) is carried out daily from two to six weeks post-surgery to maximise hip extension range of movement. Posterior hip joint capsule stretch in side-lying commences four weeks after surgery (six weeks if microfracture present) and continues until hip external rotation range is equal to the other side. Exercise sheets provided to study participants in the PT group show these in more detail [see Additional file 1].

##### Gym and aquatic program

A gym and aquatic program will commence two weeks post-surgery, following the first post-operative physiotherapy session. This will initially consist of walking in the pool and use of a stationary bike and cross-trainer, with progression to swimming and then resistance exercise for the lower body. Participants will be provided with access to local community gym and pool facilities (YMCA centres) and will be asked to carry out this aspect of the program at least twice weekly.

##### Return to sport

The physiotherapists will provide guidance to participants regarding graduated return to sport. This will include provision of functional and sport-specific drills. Therapists will be guided by a table of options arranged to suggest drills appropriate for different sporting activities. This table is provided in Additional file 2. Generally, preliminary components of sporting activity will begin six to eight weeks after surgery, and training in the actual sporting environment will commence 10 to 12 weeks after surgery.

#### Control

Participants in this group will not attend a study physiotherapist and will be requested to not undertake a formal rehabilitation program. Participants will gradually increase their physical activity levels and return to exercise and sport based on the information brochure provided.

### Physiotherapy treatment integrity

The integrity of the physiotherapy intervention will be ensured by a variety of methods. Therapist adherence to the protocol will be maximised through provision of a comprehensive treatment manual and attendance at a one-day training course outlining the study protocol and treatment program. After trial commencement, online or telephone meetings will be held to discuss any issues experienced and solutions will be instigated. Physiotherapists will use standardised, structured treatment recording forms, which will be audited by research staff. Participants will be questioned at the end of their treatment about their physiotherapy treatment experience.

### Descriptive data

Age, gender, occupation, sporting involvement, duration of hip symptoms, previous treatments, medication use, imaging and surgical findings, and surgical intervention will be obtained by questionnaire, from imaging scans and from the surgical report.

### Outcome measures

Outcome measures are summarised in Table 5.

**Table 5:** Outcome measures

**Primary outcome measures**	**Data collection instrument**	**Collection points†**
Health-related quality of life	iHOT-33	0, 14, 24 weeks
Function in sport	Sport subscale of HOS	0, 14, 24 weeks
**Secondary outcome measures**		
Symptoms, pain, function in daily living and sport, participation in physical activities hip/groin-related quality of life	HAGOS	0, 14, 24 weeks
Physical function	Activities of daily living subscale of HOS	0, 14, 24 weeks
Activity level	Modified Tegner Activity Scale	0, 14, 24 weeks
Sport participation	Heidelberg Sports Activity Scale	0, 14, 24 weeks
Global rating of overall change	Perceived overall change in hip/groin symptoms compared to baseline – 7 point ordinal scale	14, 24 weeks
**Other measures**		
Patient demographics, past treatment	Questionnaire	0 weeks
Surgical procedure	Post-surgery letter from surgeon to referring doctor	Following surgery
Adverse events	Patient logbook, questionnaire	14 weeks
Other treatments/co-interventions	Questionnaire	14, 24 weeks
Physiotherapy session attendance	Therapist treatment records	During intervention
Medication use	Questionnaire	14 weeks
Home/gym program adherence	Participant log book – number of days/times completed	Daily during intervention
	Self-rated using 11-point numeric rating scale	14 weeks

iHOT-33= International Hip Outcome Tool.

HOS=Hip Outcome Score.

HAGOS=Copenhagen Hip and Groin Outcome Score.

† 0=baseline 2 weeks pre operatively; 14 and 24 weeks are timed from the date of surgery.

#### Patient reported outcomes

The International Hip Outcome Tool (iHOT-33) is a recently developed self-administered tool that measures health-related quality of life in young active patients with hip disorders [23].This 33-item questionnaire covers four domains including: symptoms and functional limitations (16 items); sports and recreational activities (6 items); job related concerns (4 items); and social, emotional and lifestyle concerns (7 items). It uses a 100 mm horizontal visual analogue scale response format with scores ranging from 0 to 100 for each question where a higher score represents better quality of life. The overall score is calculated by taking the average out of 100 for the completed questions. The item relating to sexual activity will be omitted for ethical reasons, due to the inclusion of 16–18 year-olds in this study. For those patients who are not working for reasons other than their hip joint problems, the job-related questions are omitted and the overall score still calculated by taking the average out of 100 for the remaining questions [23]. This tool has good test retest reliability (intraclass correlation coefficient of 0.78), demonstrated face, content and construct validity and is highly responsive to clinical change (effect size of 2.0, standardised response mean of 1.7, responsiveness ratio of 6.7) [23]. In a recent comparison of the reliability of patient reported outcomes for young active adults (mean age 24 years) with FAI, we found the iHOT 33 to have a test retest reliability slightly higher than that previously estimated in an older cohort (intraclass correlation coefficient of 0.92 for total score). It is also one of the patient-reported outcomes that contain the highest number of items of particular relevance to a younger cohort, such as sports and recreation and job-related issues [24].

The Hip Outcome Scale (HOS) is a self-administered questionnaire designed to assess function in patients undergoing hip arthroscopy [1]. It assesses the degree of difficulty in performing tasks in two domains; activities of daily living (ADL, 17 items) and sport (9 items). The items are scored on a five point Likert scale from 4 to 0 with 4 being 'no difficulty’ and 0 being 'unable to do’. The score on each of the items are summed and divided by the highest potential score (68 for the ADL subscale and 36 for the sport subscale) then multiplied by 100 to obtain a percentage. In younger patients undergoing hip arthroscopy, the HOS has excellent test re-test reliability (intraclass correlation coefficient values of 0.92 for the sports subscale and 0.98 for the ADL subscale), evidence of content, construct and concurrent validity [1,25] and is responsive to clinical change (effect sizes of 1.5 and 1.2 for the Sports and ADL subscales, respectively and area under the receiver operating characteristic curves of 0.90 and 0.88) [25]. Although our reliability estimates for the HOS (ICC 0.73–0.9) [24] were lower than those reported in a slightly older (mean age 33 years) cohort of patients following hip arthroscopic surgery (ICC 0.92–0.98 [24]), in two recent systematic reviews of the clinimetric properties of patient-reported outcome questionnaires to assess hip and groin disability, the HOS was recommended by both for evaluating patients undergoing hip arthroscopy [26,27].

The Copenhagen Hip and Groin Outcome Score (HAGOS) is a newly-developed patient-reported questionnaire specifically designed for young to middle-aged, physically active individuals with hip and groin pain [27]. It is a quantitative measure of the person’s hip and groin disability according to the different levels of the International Classification of Functioning. The HAGOS consists of 37 items in six separate subscales relating to the past week, assessing pain (10 items), symptoms (7 items), physical function in daily living (5 items), physical function in sport and recreation (8 items), participation in physical activities (2 items) and hip and/or groin-related quality of life (5 items). Items are scored on a five-point Likert scale of 0 to 4, where 0 indicates no difficulty and 4 indicates extreme difficulty. A normalised score (100 indicating no symptoms and 0 indicating extreme symptoms) is calculated for each subscale. This questionnaire has been shown to have adequate measurement properties in young to middle-aged patients with chronic hip/groin pain including evidence of construct validity, responsiveness to change [27] and good to excellent test-retest reliability (intraclass correlation coefficients 0.82 to 0.91 [27] and 0.79-0.94 for the subscale [24]. Inclusion of the HAGOS will allow us to assess similarity of results from this and other self-reported measures included.

At each re-assessment time, participants will rate their perceived overall change in their hip/groin problem (compared to baseline) on a seven-point ordinal scale (much worse to much better). Scales of this kind are frequently used as an external criterion for comparison with changes in scores of other outcomes [28]. Measuring participant-perceived change using a rating of change scale has been shown to be a clinically relevant and stable method of identifying improvements that are truly meaningful from the individual perspective [29].

A modified Tegner Activity Scale and the Heidelberg Sports Activity Score will be administered to grade participants’ level of physical activity (both sport/recreation and occupational) [30,31].

#### Adherence in the physiotherapy group

The number of physiotherapy visits will be recorded for the PT group. Participants in this group will maintain a log-book to record the frequency, intensity and duration of the exercises in their home/aquatic/gym rehabilitation program. They will also rate their adherence to the home program overall, as well as to its separate components (gym program, pool sessions and home exercises), at 14 weeks post-surgery on 11-point numeric rating scales (with 0 being 'not at all’ to 10 being 'completely as instructed’).

#### Adverse events, co-interventions and medication use

Information on adverse events, co-interventions and medication use will be collected during the intervention phase using a log book in both groups as well as by self report questionnaire at 14 and 24 weeks post surgery. The CON group will record physical activity performed each week (type, duration and intensity), and specifically whether they consulted a physiotherapist following surgery.

### Sample size

The primary endpoint will be change from baseline to 14 weeks post-surgery in (i) the iHOT-33 and (ii) sport subscale of the HOS. The minimum clinically important difference to be detected in this patient population is 6.1 for the iHOT-33 [23] and 6.0 for the Sport subscale of the HOS [24]. Between-participant standard deviations have not been widely reported in the literature for these questionnaires so the study was powered to detect a moderate effect size of 0.5. Given this, the required sample for a two-tailed comparison of the two groups using analysis of covariance with baseline values as covariates, when d = 0.5, power is 0.8 and type I error is 0.05 is 41 participants per group. To allow for a 15% dropout rate a total of 100 participants will be recruited.

### Data and statistical analysis

A biostatistician will oversee the blinded analyses of the data. The primary analysis of the data will be undertaken using the principle of intention-to-treat. This analysis will include all participants including those who have missing data and those who do not fully adhere to the protocol. Some attrition is anticipated despite the fact that we will implement procedures to minimise loss to follow-up and participant withdrawal, and maximise adherence. To account for missing data, multiple imputation of missing follow-up measures, assuming missing data are missing at random and follow a multivariate normal distribution [32] will be performed as a sensitivity analysis.

Demographic and clinical characteristics as well as baseline data will be presented to assess the baseline comparability of the intervention groups. These variables will also be examined for those participants who withdraw from the study and those who remain.

Descriptive statistics will be presented for each group as the mean change (standard deviation, 95% confidence intervals) in the outcomes from baseline to each time point. For continuous outcome measures, differences in mean change will be compared between groups using linear regression random effects modelling adjusted for baseline values of the outcome. Model diagnostic checks will utilise residual plots. Similar regression models for binary and ordinal outcome measures will use random effects logistic and proportional odds models, respectively. We will also perform a per protocol analysis as appropriate.

No statistical adjustment will be made for multiple testing. All tests will be two sided and carried out at the 5% level of significance. Any changes to the study design or analysis plan will be documented with full justification.

### Timeline

Ethics approval from the Human Research Ethics Committee of the University of Melbourne was obtained in October 2012. Recruitment and training of the project physiotherapists occurred in February 2013. Recruitment of participants commenced in June 2013. All participants are expected to have completed the study by end December 2015.

## Discussion

This paper has presented the protocol for an ongoing RCT to investigate the effect of a physiotherapy-supervised rehabilitation intervention on a range of self-reported outcomes in people undergoing hip arthroscopy for management of symptomatic FAI.

The study has been designed with attention to key methodological features to minimise bias including randomisation, concealed allocation and intention-to-treat analysis. The primary outcomes are reliable and valid patient-reported measures suitable for young active individuals with FAI. The study is powered to find a moderate effect size for these outcome measures and as such smaller effects may not be detected. In addition, our study includes a longer-term follow-up. While the delivery of the physiotherapy intervention by multiple community physiotherapists risks an increase in treatment variation through the influence of therapist personality and style, it allows a more practical delivery mode that mimics clinical practice and will enhance the generalisability of the study findings.

Several authors have described rehabilitation programs following hip arthroscopy for FAI [9,10,12,33] and recommend an individualised and evaluation-based program. One of the limitations of our study design is the semi-structured nature of the physiotherapy intervention, which to some extent restricts individual tailoring of the program. However, as with all controlled trials, it is optimal that treatment variation is reduced and that the intervention is accurately reported and easily replicated. Some components of the protocol used by the authors in clinical practice have been modified or removed for this reason: the option of dry needling has been replaced by trigger point massage only, and high velocity manipulation of the lumbar spine not included, given that not all physiotherapists have the expertise or additional qualifications required to deliver these interventions.

The findings of the study will help determine whether formal rehabilitation improves outcomes following hip arthroscopy as compared to an educational brochure, and will help guide treatment decision-making.

## Competing interests

The authors declare that they have no competing interests.

## Authors’ contributions

AT, JOD, KLB and RSH conceived the project; KLB and JOD procured the project funding; KLB and RSH will co-ordinate the trial. KLB, RSH, AT, JOD, LS and DJH assisted with protocol design. AT and LS designed the physiotherapy protocol and, along with KLB, trained the physiotherapists. KLB wrote the first draft of this manuscript. MS performed the sample size calculations and designed the randomisation schedule and statistical analyses. All authors participated in the trial design, provided feedback on drafts of this paper and read and approved the final manuscript.

## Pre-publication history

The pre-publication history for this paper can be accessed here:

http://www.biomedcentral.com/1471-2474/15/58/prepub

## Supplementary Material

Additional file 1 Exercise sheets for deep hip rotator muscle strengthening. Click here for file

Additional file 2 Return to sport guidelines provided to study physiotherapists. Click here for file
