# Supplementary material for: Efficacy of a physiotherapy rehabilitation program for individuals undergoing arthroscopic management of femoroacetabular impingement – the FAIR trial: a randomised controlled trial protocol
Source: BMC Musculoskelet Disord. 2014 Feb 26;15:58. doi: 10.1186/1471-2474-15-58 (PMC3941691; doi:10.1186/1471-2474-15-58)
Supplement: Additional file 1 — Exercise sheets for deep hip rotator muscle strengthening. [file 1471-2474-15-58-S1.pdf]

Level 1 – Deep hip rotators lying face-down

<table>
<tr>
<td>

Starting position

Lie on your stomach with your knees apart.

Bend your knees so that they are at 90 degrees.

Place the sole of the foot on your non-operated leg against the inner surface of your ankle on your operated side.

Exercise

Keeping your thigh on bed, **gently** press the ankle on your operated side against the sole of the other foot.

Relax hamstrings. Relax buttock muscles. Breathe.

Place your fingers on the bony part of your bottom called the ischial tuberosity. Then move your fingers 2cm out then 2cm up.

Feel a gentle contraction of QF muscle underneath your fingers. Hold for 3 seconds then relax for 2 seconds (approximately 12 repetitions in one minute)

Dosage
Pre surgery: 1 minute at least 6 times per day
After surgery: 1 minute, 3-4 times per day

Indicators for progression

When able to do 12 contractions in one minute with good technique

</td>
<td>

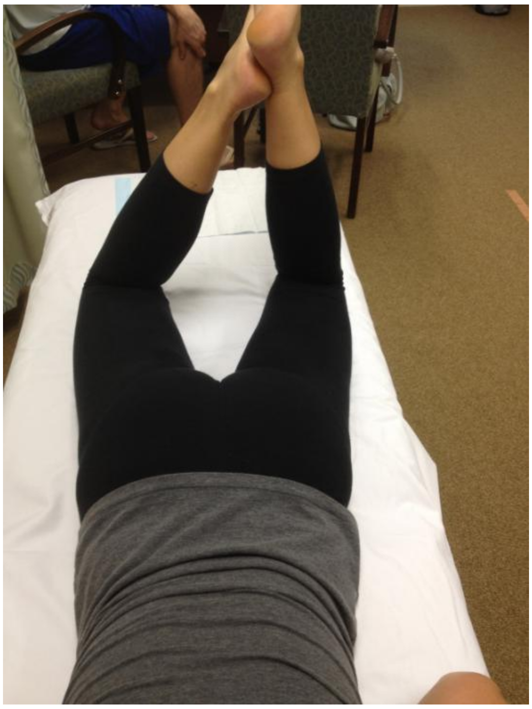

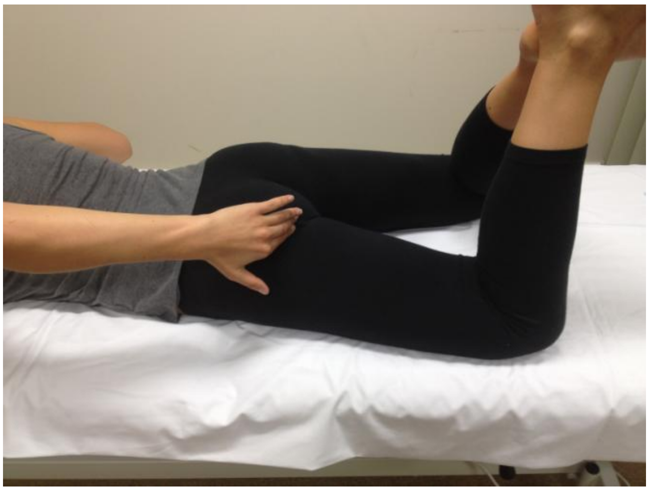

</td>
</tr>
</table>

Level 2 – Deep hip rotators on all fours

**Starting position**<br>4 point kneeling with the lower back in a neutral position.<br>**Exercise**<br>Relax hamstrings. Relax buttock muscles. Breathe.<br>Place your fingers on the bony part of your bottom called the ischial tuberosity. Move your fingers 2cm out then 2cm up.<br>Feel a gentle contraction of QF muscle underneath your fingers. Hold for 3 seconds then relax for 2 seconds.<br>It may help to think of pulling your thigh up towards your pelvis.<br>**Dosage**<br>*Pre surgery*: 1 minute, at least 6 times per day<br>*After surgery*: 1 minute, 3-4 times per day<br>**Indicators for progression**<br>When able to do 12 contractions in one minute with good technique<br>**Variations:**<br>If shoulder pain or fatigue are a problem, support your chest on the seat of a chair	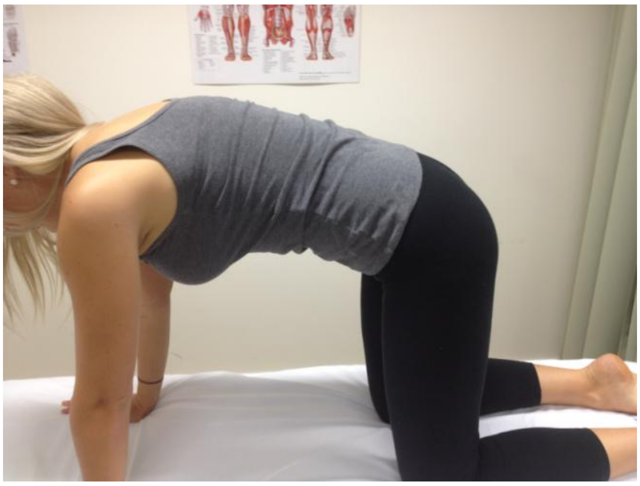

Level 3 - Deep hip rotators on hands and knees with hip external rotation (twisting foot inwards)

Starting position

4 point kneeling with the lower back in a neutral position.

The operated leg should be parallel to the opposite side, in a relaxed position (ie not twisted inward or outward)

Exercise

Activate QF muscle, and then gently rotate the foot on the operated leg inwards. Aim for your foot to be above your calf on your non-operated side at the end of the movement.

Slowly return to the starting position.

Continue to hold the QF contraction whilst performing a total of 5 repetitions of the movement. Rest for 3 seconds then repeat, continuing for one minute.

Dosage

One minute, 3-4 times per day

Indicators for progression

When able to perform exercise for one minute maintaining good technique and with no pain.

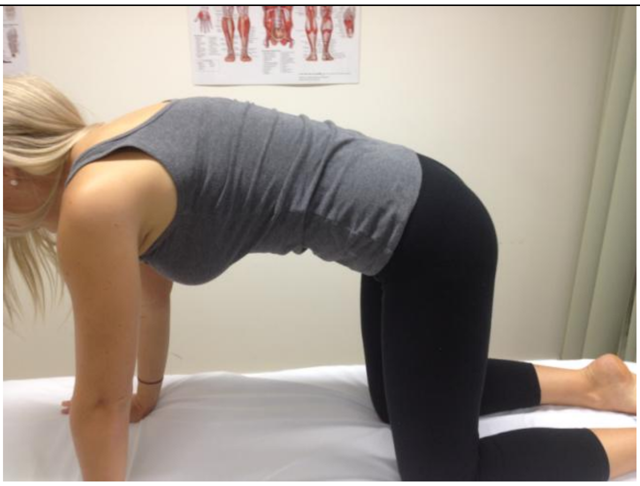

Starting position

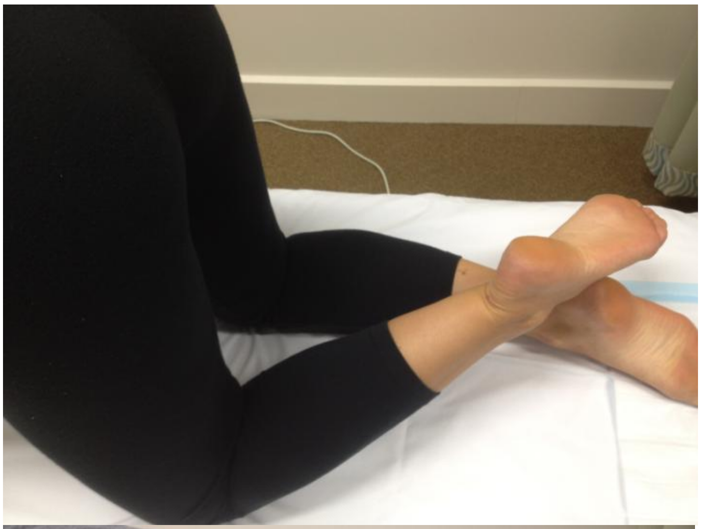

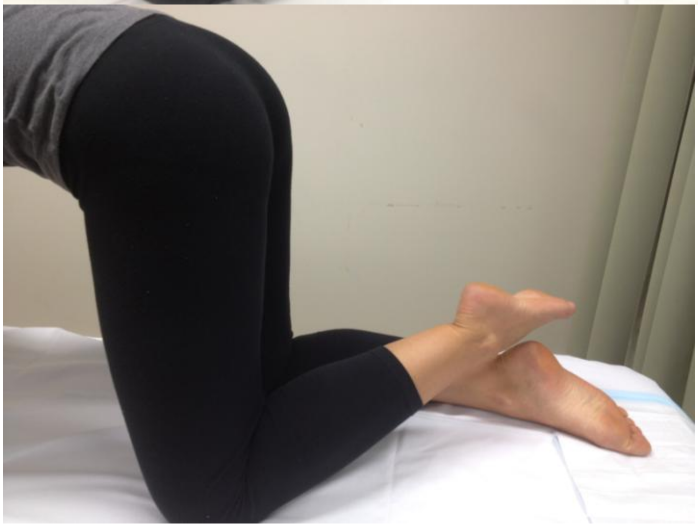

External rotation, twisting thigh to take foot inwards

Level 4 - Deep hip rotators in 4-point-kneeling with hip internal rotation (twisting foot outwards)

Starting position

4 point kneeling with the lower back in a neutral position.

The operated leg should be parallel to the opposite side, in a relaxed position (ie not twisted inward or outward)

Relax hamstrings. Relax gluteals. Continue to breathe throughout the exercise.

Exercise

Activate QF muscle on the operated side. Keep the muscle tightened while you gently rotate the foot on the operated leg outwards. Take the foot as far as you can go comfortably.

Slowly return to the starting position.

Continue to hold the QF contraction whilst performing a total of 5 repetitions of the movement. Rest for 3 seconds then repeat, continuing for one minute.

Dosage

5 reps then rest, repeat for one minute

Indicators for progression

When able to perform exercise for one minute maintaining good technique and with no pain.

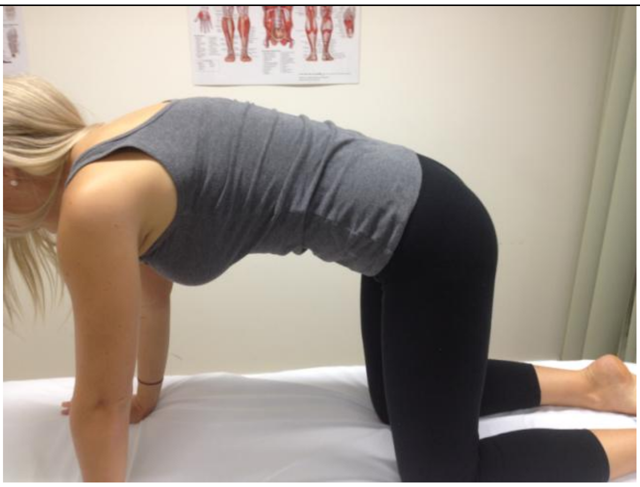

Starting position

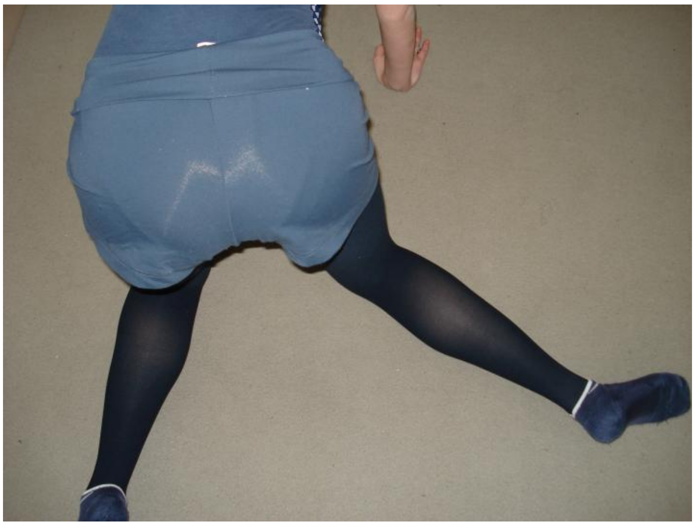

Internal rotation, twisting thigh to take foot outwards

Level 5, Exercise 1 - Hip external rotation on hands and knees with resistance band (Theraband®)

Starting position

Kneel on all fours with your lower back in a neutral position.

Your operated leg should be parallel to the opposite leg, in a relaxed position (ie not twisted inward or outward).

Place resistance band around your ankle on your operated side as shown.

Exercise

Activate the QF muscle on your operated side. Gently twist your leg to take your foot on the operated side inwards against the pull of the elastic band. Aim for your foot to be above your calf on your non-operated side at the end of the movement.

Slowly return to the starting position, carefully controlling the speed of the movement.

Rest for 3 seconds then repeat, continuing this process for one minute.

Dosage

One minute, 3-4 times per day

Indicators for progression

Able to perform exercise for one minute pain-free; able to maintain good technique, with smooth, controlled movement.

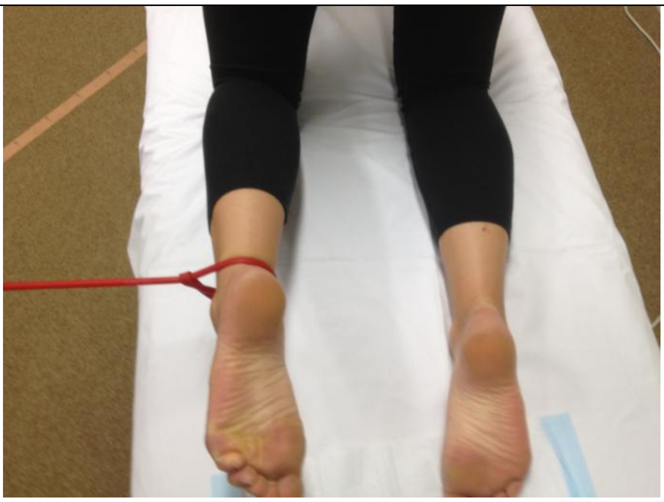

External rotation – starting position

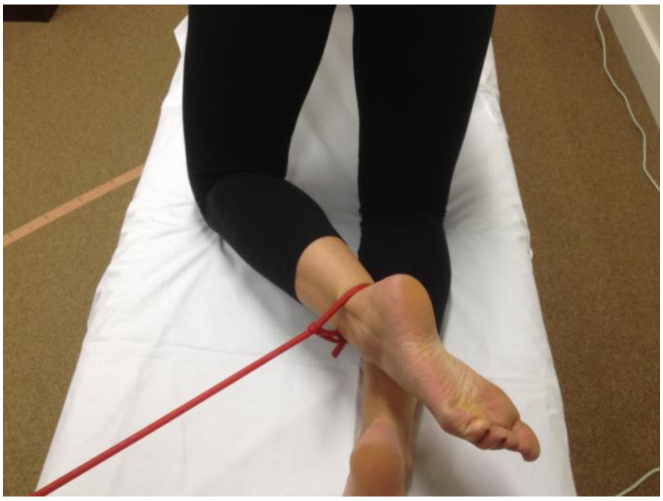

External rotation exercise

Resistance band:

NONE YELLOW RED GREEN

Level 5, Exercise 2 - Hip internal rotation on hands and knees with resistance band (Theraband®)

Starting position

Kneel on all fours with your lower back in a neutral position.

Your operated leg should be parallel to the opposite leg, in a relaxed position (ie not twisted inward or outward).

Place resistance band around your ankle on your operated side as shown.

Exercise

Activate the QF muscle on your operated side. Keep the muscle tightened. Gently twist your leg to take your foot outwards, away from your other leg, against the pull of the elastic band. Take your foot as far as you can go comfortably.

Slowly return to the starting position, carefully controlling the speed of the movement.

Rest for 3 seconds then repeat, continuing this process for one minute.

Dosage

One minute, 3-4 times per day

Indicators for progression

Able to perform exercise for one minute pain-free; able to maintain good technique, with smooth, controlled movement

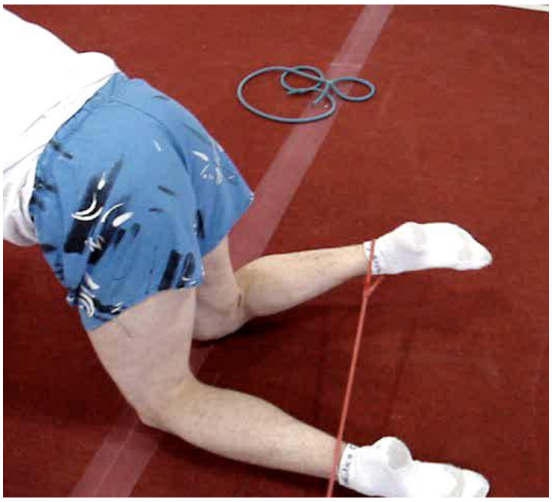

Internal rotation exercise

Resistance band:

NONE YELLOW RED GREEN

Level 6, Exercise 1 - Hip external rotation with resistance (Theraband®) and abduction with belt on hands and knees

Starting position

Kneel on all fours with your lower back in a neutral position and your knees about two fist-widths apart.

Place a Pilates belt or resistance band around both your thighs and a resistance band around your ankle on your operated side as shown.

Exercise

Keep your knee on the bed. Tighten the muscle at the side of your hip by pushing your operated side against the belt (as though trying to take your knees apart).

At the same time, activate the QF muscle on your operated side. Gently twist your leg to take your foot inwards against the pull of the elastic band. Aim for your foot to be above your calf on your non-operated side at the end of the movement.

Slowly return to the starting position, carefully controlling the speed of the movement.

Rest for 3 seconds then repeat, continuing this process for one minute.

Dosage

One minute, 3-4 times per day

Indicators for progression

Able to perform exercise for one minute pain-free; able to maintain good technique, with smooth, controlled movement.

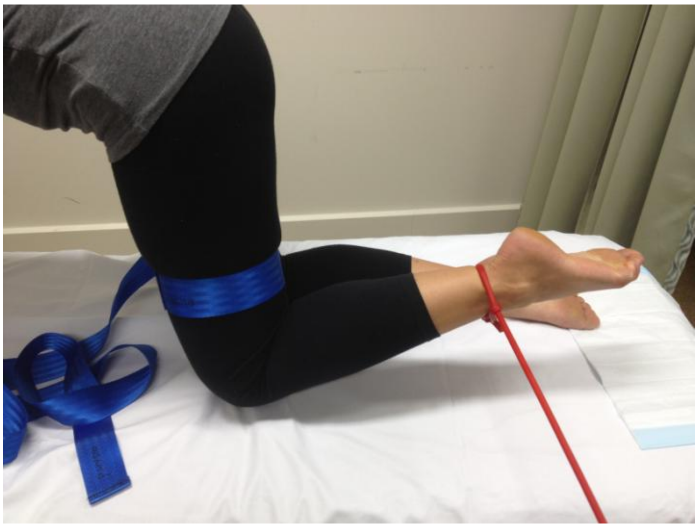

External rotation – Red Theraband® resisting twisting movement and blue Pilates belt providing resistance to thigh movement

To be done in conjunction with Level 6, Exercise 2 (internal rotation)

Resistance band:

NONE YELLOW RED GREEN

Level 6, Exercise 2 - Hip internal rotation with resistance (Theraband®) and abduction with belt in 4-point-kneeling

<table>
<tr>
<td>

Starting position

Kneel on all fours with your lower back in a neutral position and your knees comfortably apart.

Place a Pilates belt or resistance band around both your thighs and a resistance band around your ankle on your operated side as shown.

Exercise

Keep your knee on the floor. Tighten the muscle at the side of your hip by pushing your operated side against the belt (as though trying to take your knees apart).

Activate the QF muscle on your operated side. Gently twist your leg to take your foot outwards, away from your other leg, against the pull of the elastic band. Take your foot as far as you can go comfortably.

Slowly return to the starting position, carefully controlling the speed of the movement.

Rest for 3 seconds then repeat, continuing this process for one minute.

Dosage

One minute, 3-4 times per day

Indicators for progression

Able to perform exercise for one minute pain-free; able to maintain good technique, with smooth, controlled movement.

</td>
<td>

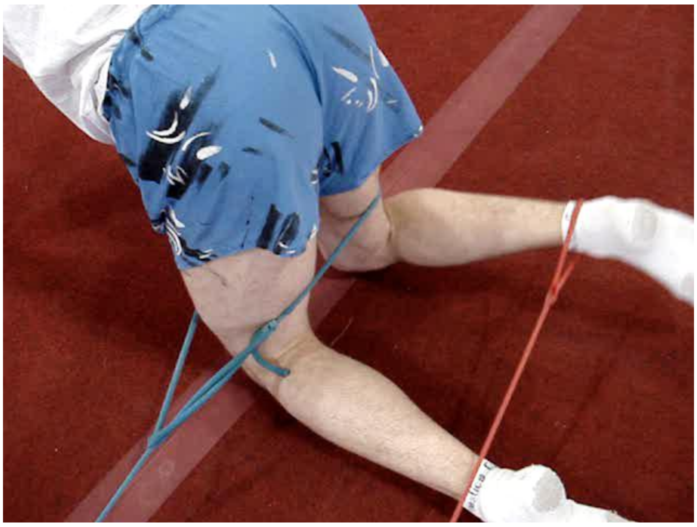

Internal rotation – Red Theraband® resisting twisting movement and blue Theraband® providing resistance to thigh movement

To be done in conjunction with Level 6, Exercise 1 (external rotation)

Resistance band:

NONE YELLOW RED GREEN

</td>
</tr>
</table>

Level 7, Exercise 1 - Arabesque

**<u>Starting position</u>**<br>Standing with feet shoulder width apart.<br>**<u>Exercise</u>**<br>Tense the QF muscle on your operated side. Lean your chest forward and take your arms out to the sides as you lift the non-operated side behind you.<br>Hold the position for 3 seconds. Bring your non-operated leg down to take a step forward.<br>Step forward on to your operated side and repeat.<br>**<u>Dosage</u>**<br>Do as many repetitions as you are able to with good technique and no pain. Aim to do 26 repetitions in a row. You may break this up in the initial stages, for example 5 arabesques, 5 times per day.<br>**<u>Indicators for progression</u>**<br>Able to complete 26 repetitions in a row with good technique, and no pain.	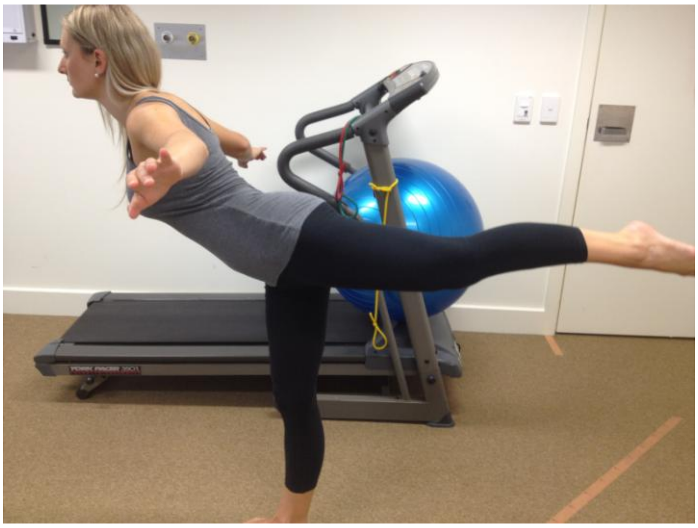

Level 7, Exercise 2 - Duck Walk

<table>
<tr>
<td>

Starting position

Have your feet shoulder-width apart.

Place a Pilates belt (or other belt) around your thighs, just above your knees. It is best to use a belt rather than exercise band for this exercise.

Do a ¼ squat, so that your knees are bent to around 30 degrees.

Your knees should be over your big toes.

Place your fingers on your QF muscle to check for/improve contraction.

Exercise

Keep your ¼ squat position as you walk forward 10 metres.

Keep your QF tense during the exercise.

Dosage

Do one walk of 10 metres, 10 times per day.

Indicators for progression

Able to complete 10 metre walk, with good technique and no pain.

</td>
<td>

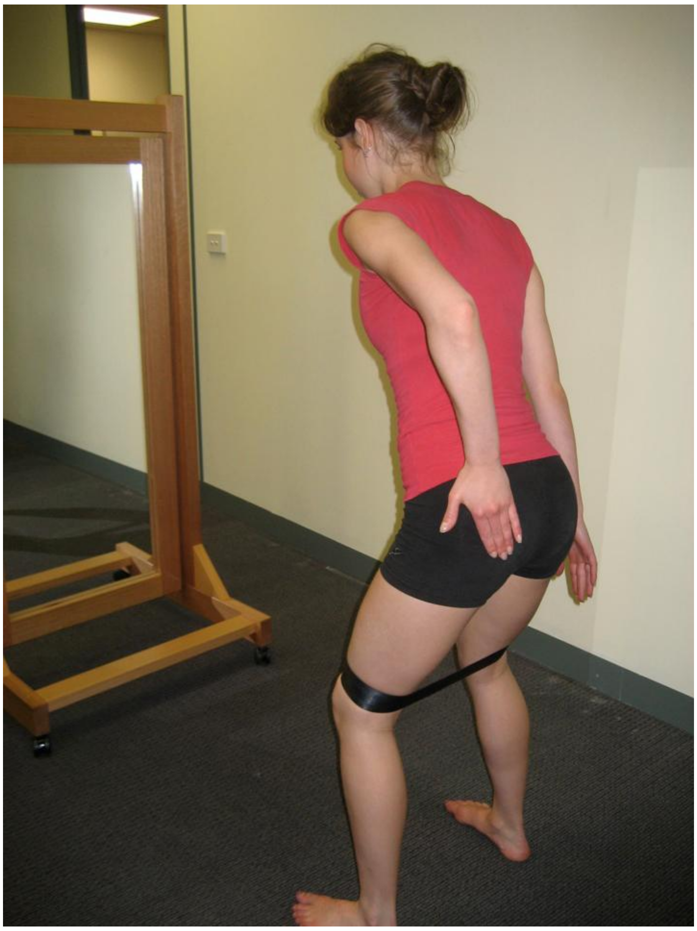

</td>
</tr>
</table>

Back of hip (posterior capsule) stretch

Starting position

Lie on your non-operated side.

Place a folded towel near the edge of the bed as shown in the picture.

Exercise

Bend your operated hip up as close to a right angle as is comfortable. Roll your hip forward and drop your knee over the edge of the bed.

Dosage

Take the stretch to the point of tension (not pain) and hold for 30 seconds. Do this three times.

Indicators for progression

Continue this exercise daily until the external rotation movement (dropping your knee out to the side when lying on your back) is equal to your non-operated hip

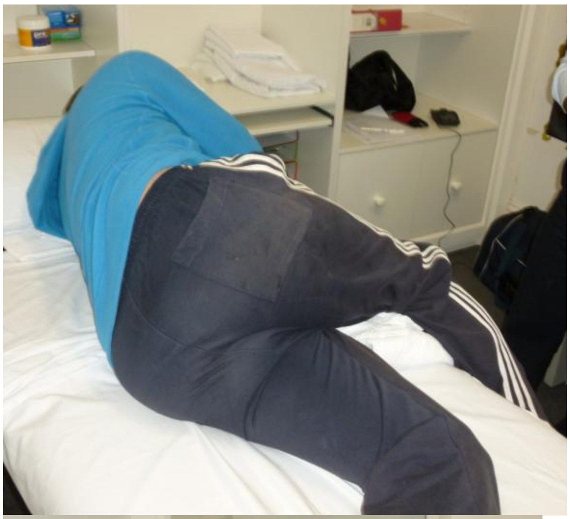

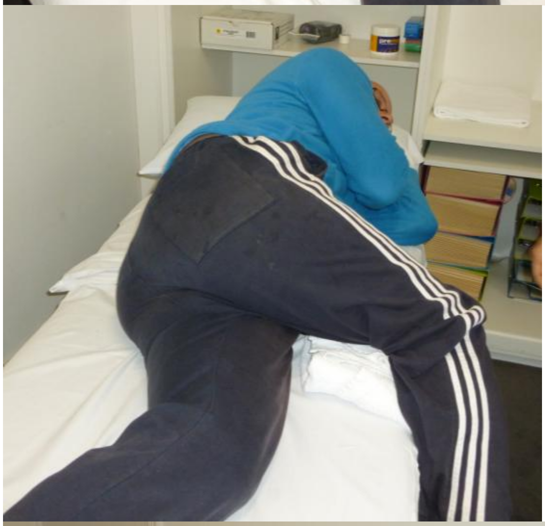

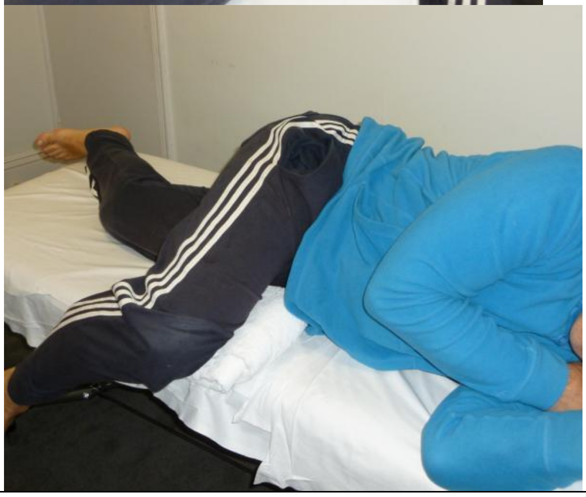

Front of hip (anterior capsule) stretch

<table>
<tr>
<td>
<u>Starting position</u>
Lying on your back on a bed. Bend up your non-operated leg.
Your bottom should be near the edge of the bed on your operated side.
<u>Exercise</u>
Take your operated leg out to the side and allow it to drop over the edge of the bed. Your thigh should still be supported by the bed.
You should feel a stretch in the front of your hip.
<u>Dosage</u>
5 minutes first thing in the morning, every day.
<u>Indicators for progression</u>
This exercise should be performed daily from two weeks after surgery until six weeks after surgery
</td>
<td>
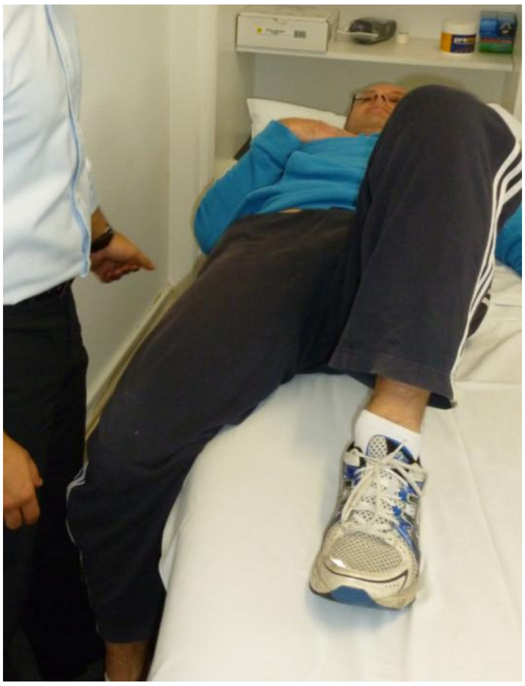
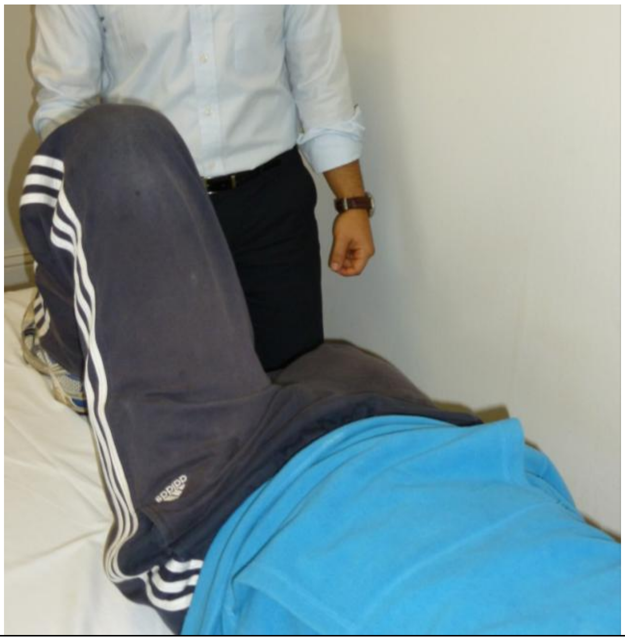
</td>
</tr>
</table>

Pendulum hip exercises in 4 point kneeling

<table>
<tr>
<td>
<u>Starting position</u>

Kneel on all fours with your lower back in a comfortable, neutral position and your head/neck comfortably relaxed.
</td>
<td>
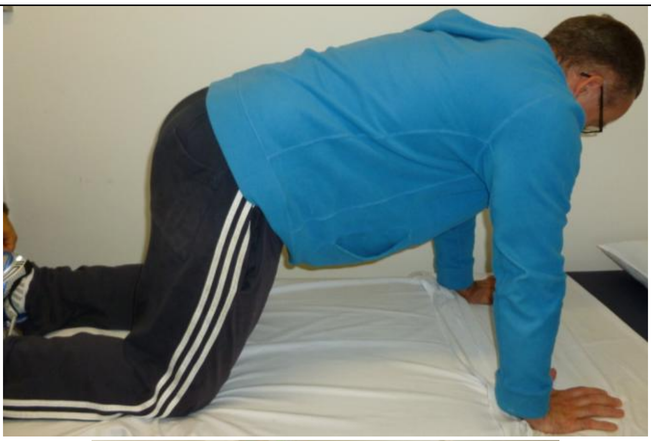
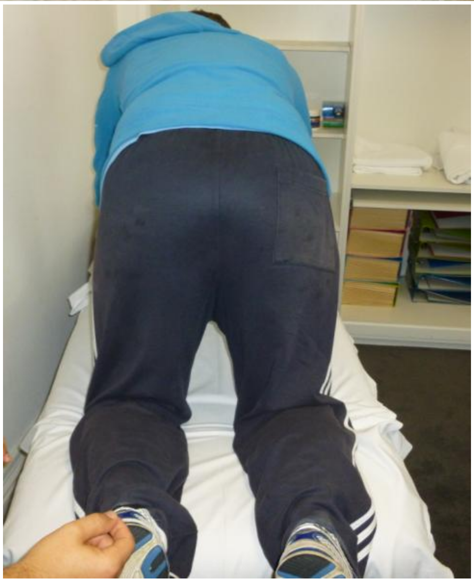
Starting position
</td>
</tr>
</table>

Exercise

Shift most of your weight onto your hands and your non-operated knee. Only a little weight should go through your operated side.

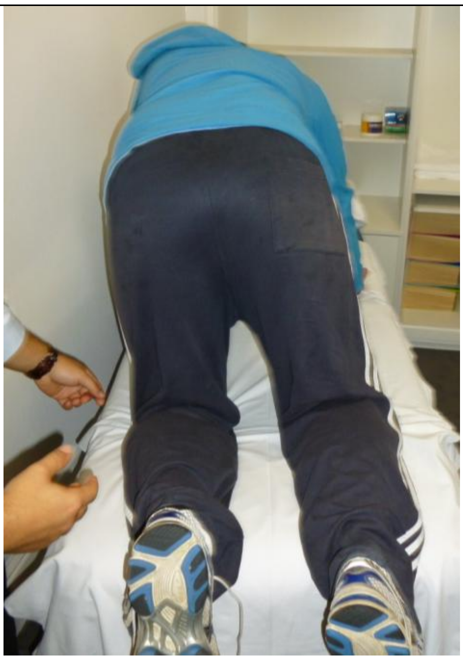

Shift weight onto your operated side

Gently slide the knee on your operated side along the bed/floor towards your hands as far as comfortable. Then slide it back down the bed as far as comfortable.

Imagine that your leg is hanging and slowly swinging back and forth like a pendulum.

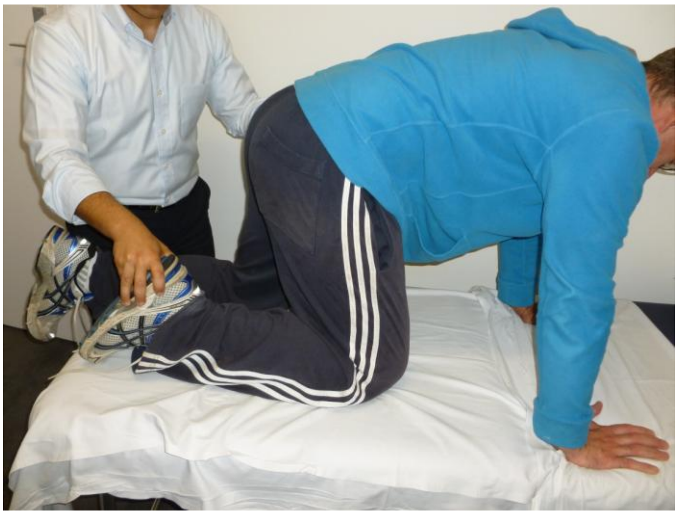

Slide operated leg forward as far as comfortable

Dosage

Smooth and slow repetitions for one minute

Indicators for progression

Once you have full pain-free movement of hip flexion (bringing your knee up towards your chest) you may stop including this exercise in your program

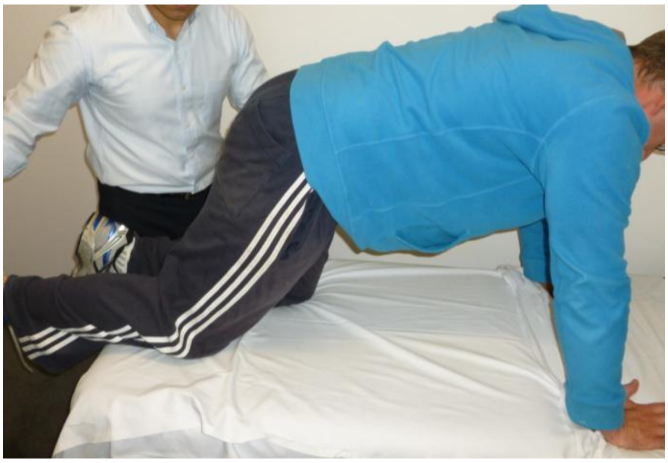

Slide your operated leg back as far as comfortable
