# Supplementary material for: Efficacy of a physiotherapy rehabilitation program for individuals undergoing arthroscopic management of femoroacetabular impingement – the FAIR trial: a randomised controlled trial protocol
Source: BMC Musculoskelet Disord. 2014 Feb 26;15:58. doi: 10.1186/1471-2474-15-58 (PMC3941691; doi:10.1186/1471-2474-15-58)
Supplement: Additional file 2 — Return to sport guidelines provided to study physiotherapists. [file 1471-2474-15-58-S2.docx]

**Additional file 2: Return to sport guidelines provided to study physiotherapists**

**Football**

| **Technique** | **Dosage** | **Time-frames** | |
| --- | --- | --- | --- |
| Running drills around oval– initially ½ jogging and ½ running | Start with 6 laps and increase by two laps per week.  Aim for 8-12 laps prior to discharge | Starting week 6 |  |
| “Zig-zag jogging” | Variable distance depending on position played.  Speed should be 50%, then 75% then 100%. May use RPE scale to rate exertion | Starting weeks 6 -10 |  |
| Kicking drills – distance | 10 metres for 15mins  20 metres for 10mins  30-40 metres for 5mins | Week 8 -12 |  |
| Kicking drills – on the run | As for distance kicking drills | Week 10 – 12 |  |
| Hand ball drills - All directions and with variable degree of impact. | Aim to manage 15 minutes prior to discharge | Week 8 -10 |  |
| Marking drills from short and long kicks | 15 mins | Week 8-12 |  |
| “Beep test” | Progress to be at pre-surgical level, Rating Perceived Exertion scale “hard” | Week 10 |  |
| Tackling drills – into bags initially then progress to full body tackling | 10-15 mins | Full body tackling by week 11 post surgery |  |

**Tennis**

| **Technique** | **Dosage** | **Time-frames** |
| --- | --- | --- |
| Foot drills on court | 15-20mins initially, then progress length of time | May commence in week 6 if strength allows (ie able to manage double elastic band deep hip rotators in 4 point kneel. Otherwise wait til wk 8 post surgery |
| Forehand drills | 15-20mins initially, then progress length of time | Week 8 post-surgery |
| Backhand drills | 15-20mins initially, then progress length of time | Week 8 post-surgery |
| Serving practice | 10 minutes of serving on court | Commence Week 8 |
| Increasing speed of hitting | Commence at 75% (Week 8-11) then progress to 100% | Commence Week 8 |
| Cross court drills | Full load | Week 8 – 12 |

**Grass hockey**

| **Technique** | **Dosage** | **Time-frames** |
| --- | --- | --- |
| Running drills on oval | ½ running and ½ jogging  Begin with 6 laps and progress to 14 laps | Commence Week 8 and increase to discharge |
| Change of direction drills/ zig-zag drills on the field | Progress from 50% of maximum to 100% or easy to very hard on RPE | Week 8- 10 |
| Corner hit-outs | 10 mins initially, then progressing to 15 mins | Commence Week 8 |
| Acceleration/deceleration and tackling drills with ball on field | 15-20 minutes | Week 10 |

**Ballet, gymnastics and dance**

It is critically important that deep hip muscle control is excellent in 4-point-kneel, arabesque and ¼ squat positions before loading further.

| **Technique** | **Dosage** | **Time-frames**  **(post-surgery)** |
| --- | --- | --- |
| Bar work | 30 mins, 3 times per week | Commence Week 7, 8 or 9 depending on control |
| Basic jumps (jeté) | 10 mins practice, 3x times per week | Commence Week 8-10 |
| Jeté Grand  (Must be strong in ALL QF exercises first) | 15 mins practice, 3 times per week | Commence Week 8-10 |
| Pirouette – half | 20 repetitions,  3 times per week | Commence Week 8 |
| Pirouette – full | 20 reps,  3 times per week | Commence Week 9 – 10 |
| Plié – control must be emphasised | Commence at 10, 3 times per week and increase as appropriate | Commence Week 8 – 10 |
| Pointe | 20 mins per week | Commence Week 10 |
| Parallel Bar | No split work. 10 mins, 3 times per week | Commence Week 6 |
| High Bar and Rings | No dismount. 10 mins, 3 times per week | Commence Week 6 |
| Basic floor work (Pommel horse and vault) | No tumbling. 10 mins, 3 times per week | Commence Week 10 |
| Trampoline tricks | Commence at 10 mins, 3 times per week and increase as appropriate | Commence Week 8 – 10  (e.g. Week 10 if microfracture) |
